# Supplementary material for: Astrovirus infects actively secreting goblet cells and alters the gut mucus barrier
Source: Nat Commun. 2020 Apr 29;11:2097. doi: 10.1038/s41467-020-15999-y (PMC7190700; doi:10.1038/s41467-020-15999-y)
Supplement: Supplementary file 1 — Supplementary Information [file 41467_2020_15999_MOESM1_ESM.pdf]

Astrovirus infects actively secreting goblet cells and alters the gut mucus barrier

Cortez et al.

Supplementary Information

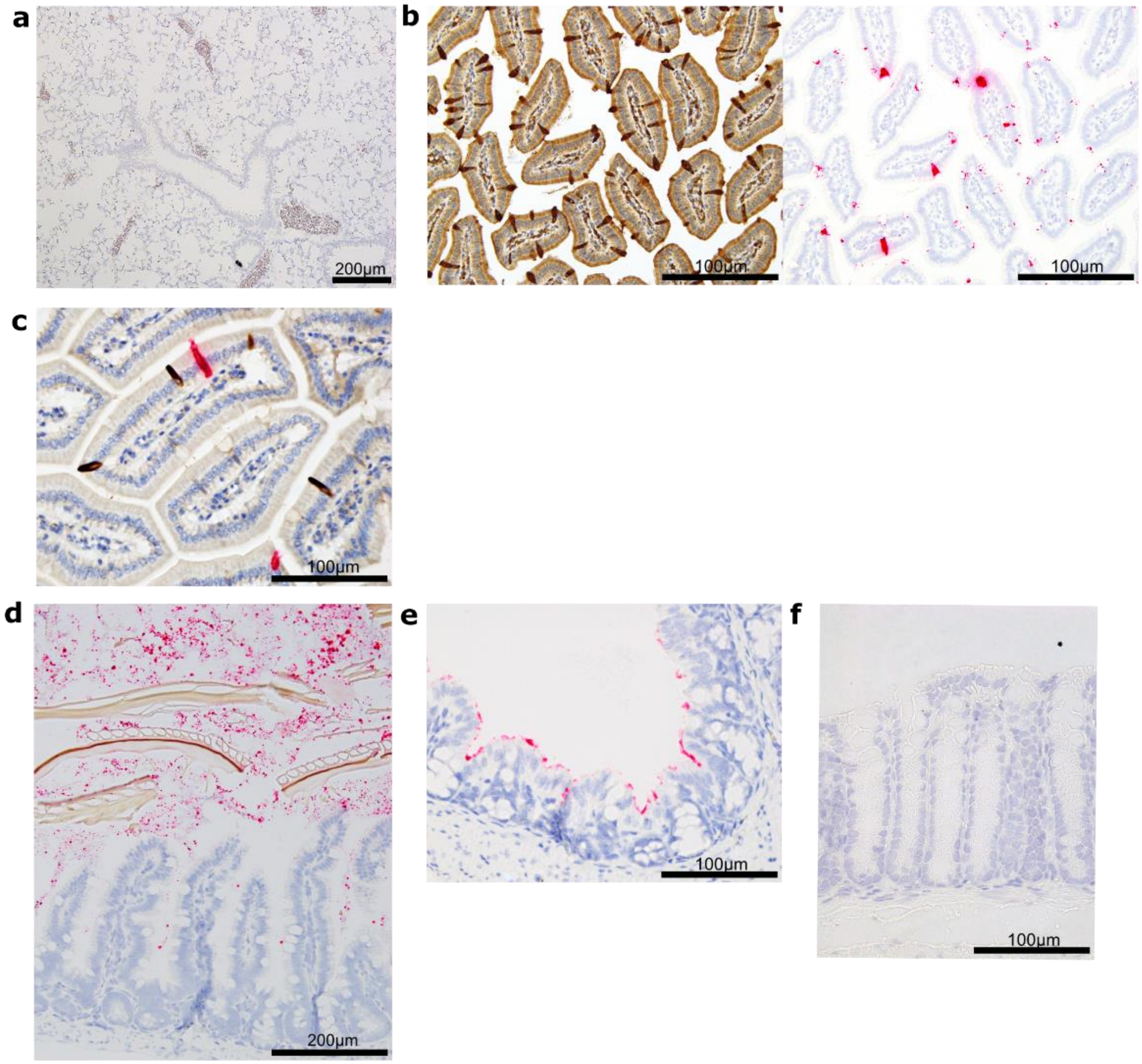

Supplementary Figure 1. Hybridization of murine astrovirus-specific probes. a A lack of staining was detected in tissues without active replication, such as the lung of infected animals (n=4). **b** Serial sections from infected animals (n=5) stained with the goblet cell marker, MUC2 (brown) overlaps with the in situ hybridization probe (red), unlike **c** co-staining with the tuft cell marker, DCLK-1 (brown) from n=2 animals. **d** Virus (red) in luminal contents of ileum. **e** Virus detected within the mucus layer above colonocytes in the proximal, but not distal colon of n=5 animals **f**. Representative images are from tissues collected at 10 dpi. Date shown are from 2 replicate experiments.

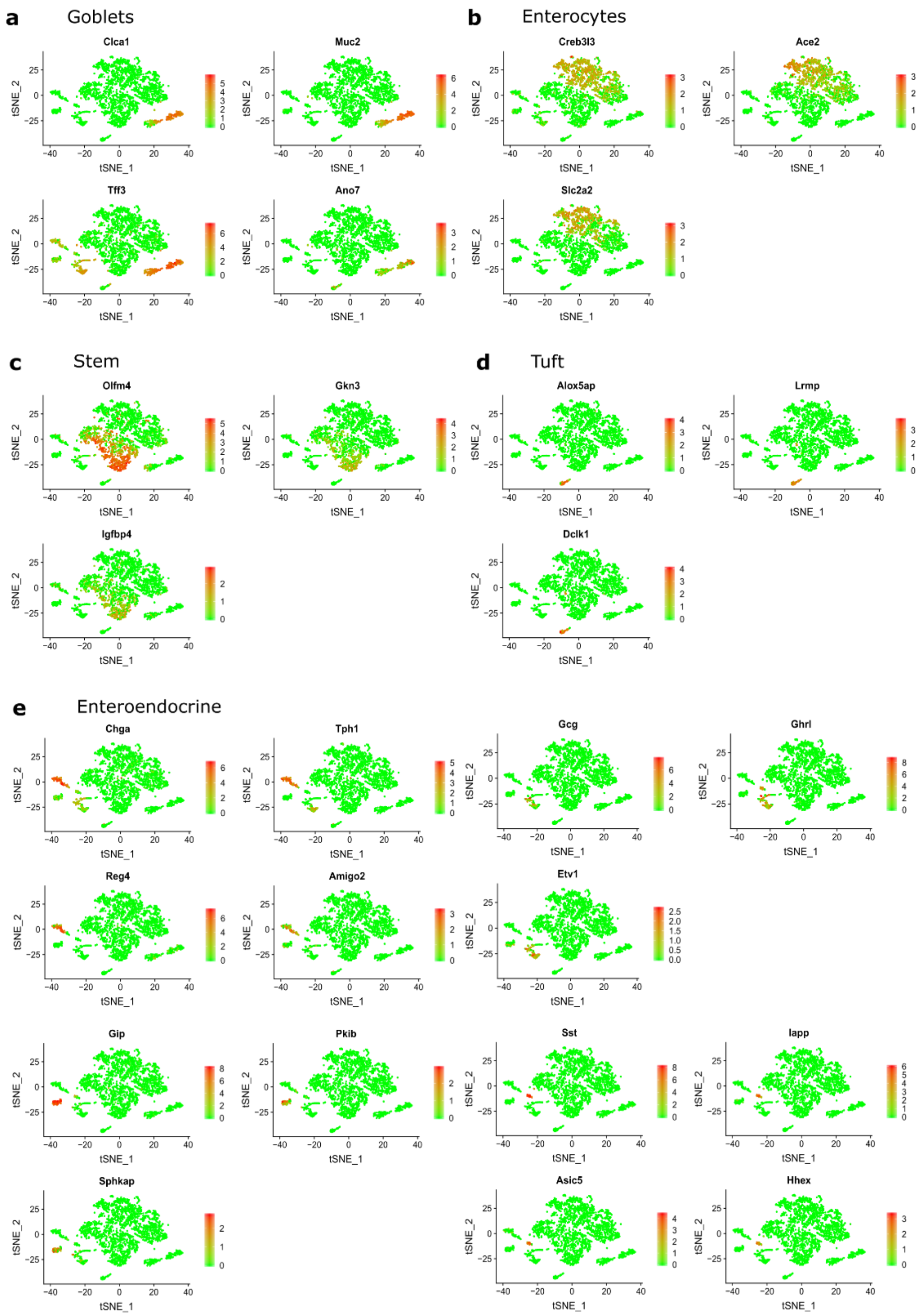

Supplementary Figure 2. Transcriptional profiling of gut epithelial subsets. Aggregated data of all duodenal epithelial cells (n= 2,973 individual cells) from both infected and uninfected (n=4 mice/group) as represented by t-SNE clustering and colored according to gene expression. Unique cell clusters identified by Seurat were classified based on the top 2-4 distinguishing genes expressed within those clusters, including **a** Goblet, **b** Enterocyte, **c** Stem, **d** Tuft, and **e** Enteroendocrine cells. Notably, paneth cells were not an identifiable cluster due to our villi-targeted isolation that did not enrich for cells that reside in the crypt.

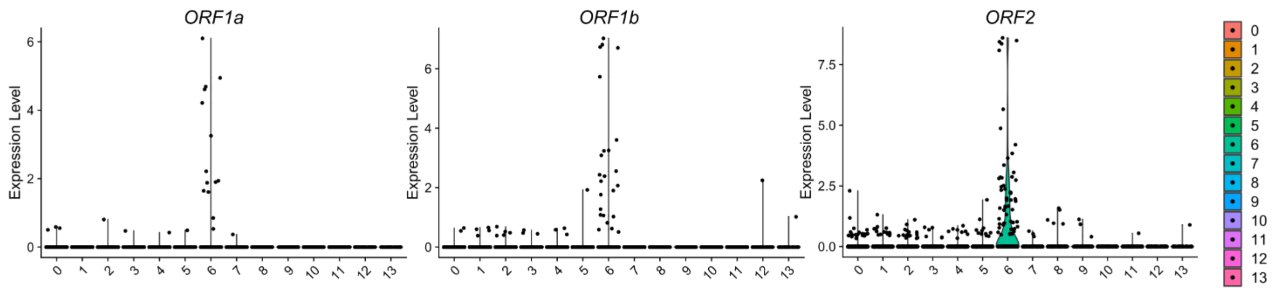

Supplementary Figure 3. Low level detection of murine astrovirus in non-goblet cell populations. Transcripts of open reading frames (ORF) 1a, 1b, and 2 of murine astrovirus detected in enterocyte (clusters 0, 1, 5), stem (clusters 2-4), enteroendocrine (clusters 8-10, 13), tuft (cluster 12), and goblet cells (clusters 6, 7). Notably, only goblet cells expressed transcripts of all 3 ORFs. Colors in legend correspond to those shown in tSNE plot in Fig. 2a.

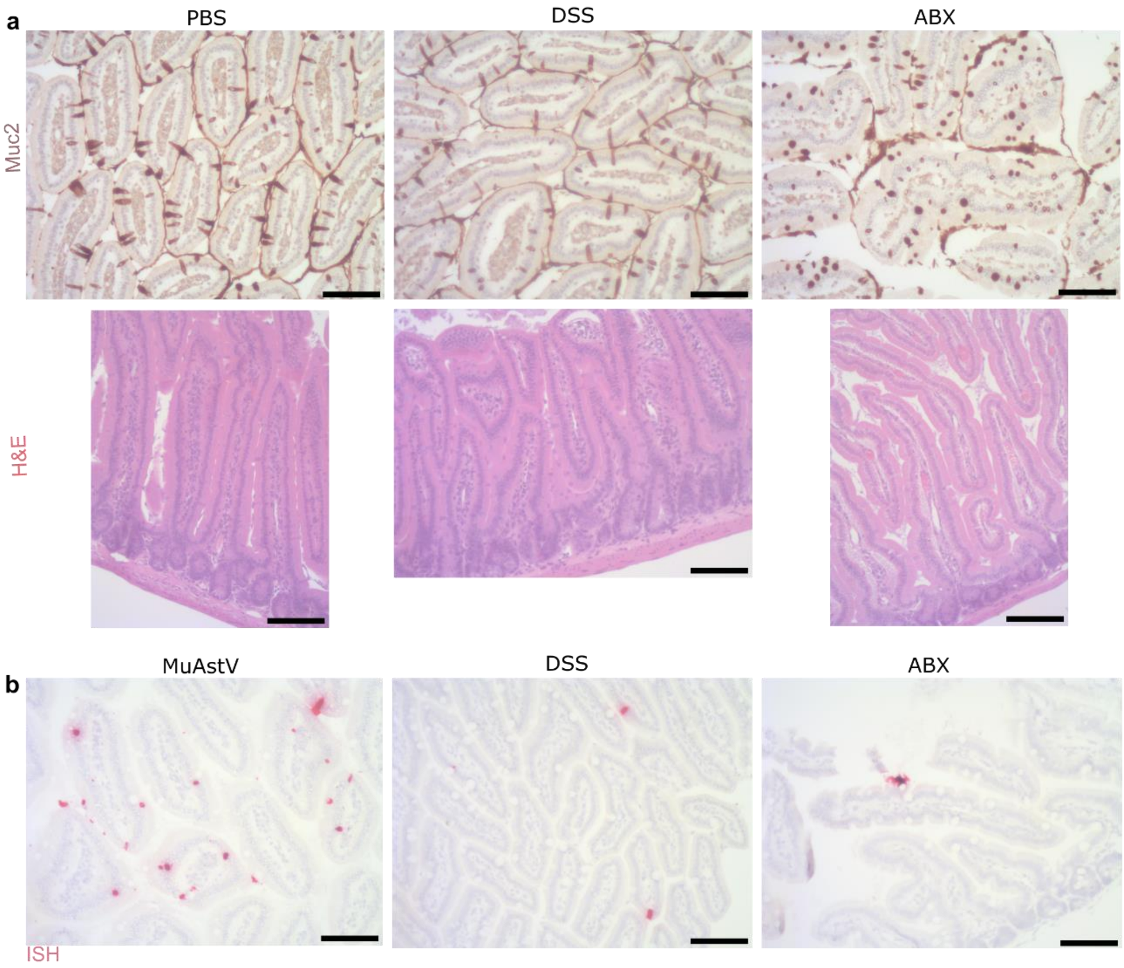

Supplementary Figure 4. Histologic findings after drug treatments that altered goblet cell physiology. Representative images from groups of 8-week-old mice treated with PBS (n=4), 2% DSS for 1 day prior to infection (n=8) or broad-spectrum antibiotics (ABX) for 7 days prior to infection (n=8). **a** Tissues were harvested on day 6 post-infection and stained for Muc2 and H&E to denote altered goblet cell morphology and tissue architecture in comparison to PBS-treated animals. **b** Consistent with qRT-PCR results in Fig. 3a and b, both treatments (n=8/group) resulted in reduced virus staining by ISH in comparison to untreated murine astrovirus-infected animals (n=5). Data shown are from 2 replicate experiments. Scale bar = 200μm

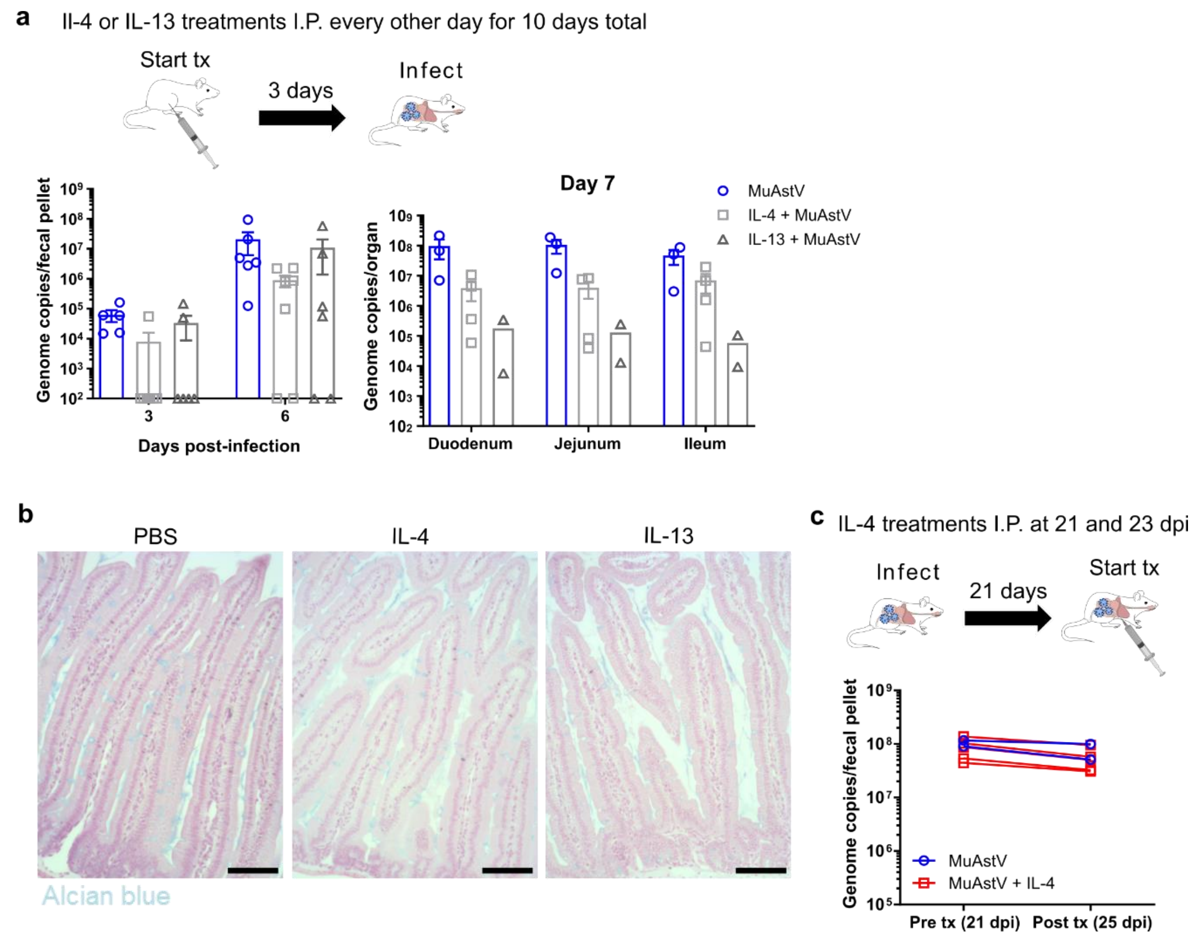

Supplementary Figure 5. IL-4 and IL-13 treatments fail to induce goblet cell hyperplasia or mucus secretion. a 8-week-old mice were treated with IL-4 immune complex, IL-13, or vehicle (PBS) via intraperitoneal injection every other day for 10 days (-3, -1, 1, 3, and 5 dpi). Only 1 out of the 7 IL-4 treated mice (IL-4 + MuAstV) had detectable virus in the feces at 3 dpi, with 5 out of 7 finally shedding virus by 6 dpi. Similarly, only 2 of the 6 IL-13 treated (IL-13 + MuAstV) mice had detectable virus in the feces at 3 dpi, with 4 out of 6 finally shedding virus by 6 dpi. In contrast, all untreated mice (MuAstV; n=5) shed virus at 3 dpi and 6 dpi. These trends were consistent with lower virus levels detected in the duodenum, jejunum, and ileum of treated mice (n=3/group) in comparison to untreated mice (n=2) at 7 dpi. Mean and SEM are noted when possible. Y-axes are drawn at the lower limit of detection for the qRT-PCR assay. Data shown are from a single experiment. **b** Reduced staining of secretory cells via alcian blue in treated (n=3/group) versus untreated/uninfected mice (n=2) 7 dpi. Scale bar = 200μm **c** 8-week-old mice were treated with IL-4 (n=5) or vehicle (PBS) (n=2) via intraperitoneal injection on days 21 and 23 dpi. Treated mice shed comparable levels of virus as untreated mice in the feces collected pre- and post-treatment. Source data are provided as a Source Data file.

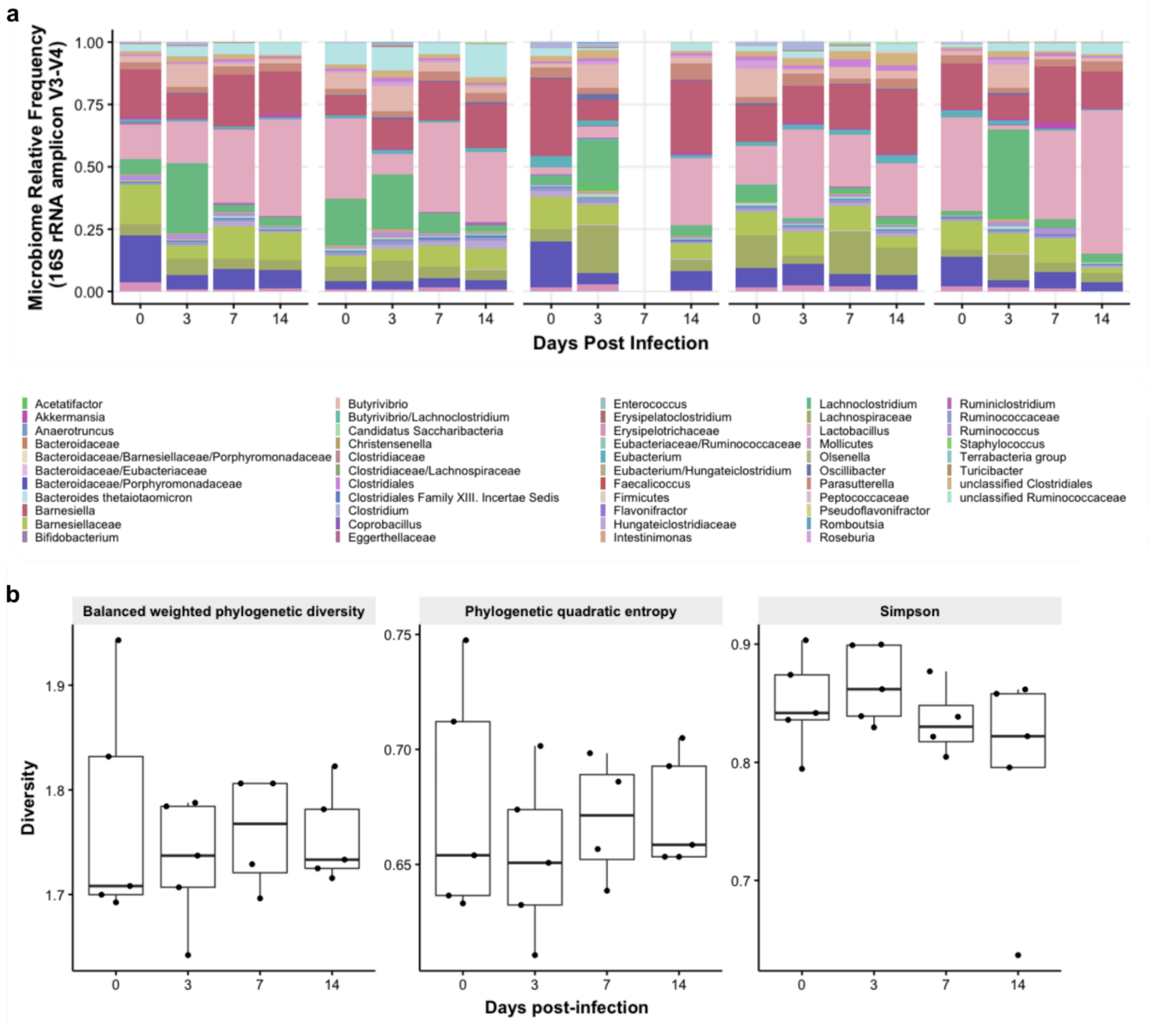

Supplementary Figure 6. Fecal microbiome composition and diversity after murine astrovirus infection. Fecal pellets were collected at indicated time points from n=5 mice and examined by 16S rRNA amplicon sequencing of V3-V4. **a** Overall composition of the fecal microbiome changes minimally throughout infection. Day 7, mouse 3 feces data was discarded due to a failed sequencing run that produced too few reads for analysis. **b** Three microbial diversity indices showed that diversity did not significantly change during infection. Box plots represent the mean and upper/lower quartiles. Data shown are from a single experiment.

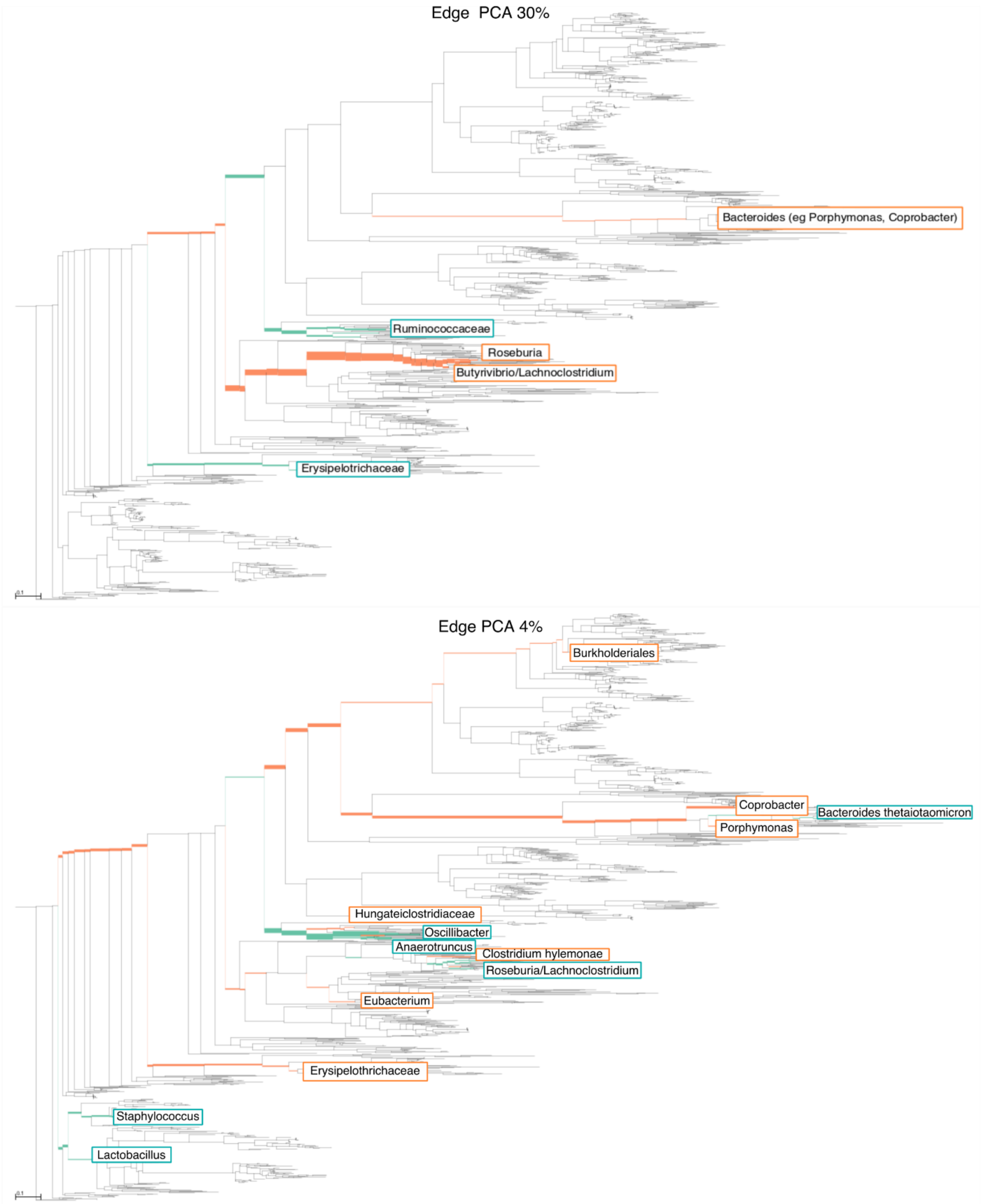

Supplementary Figure 7. Murine astrovirus-associated changes to mucus barrier altered the microbiome composition. Edge principal components analysis identifies phylogenetic edges that increase (marked in orange on the tree) and decrease (marked in blue on the tree) in the principal components that correspond to 30% (y-axis in Figure 4C) and 4% (x-axis in Figure 4C) of sample variation. The edges thickness is proportional to the magnitude of contribution to the principal component.

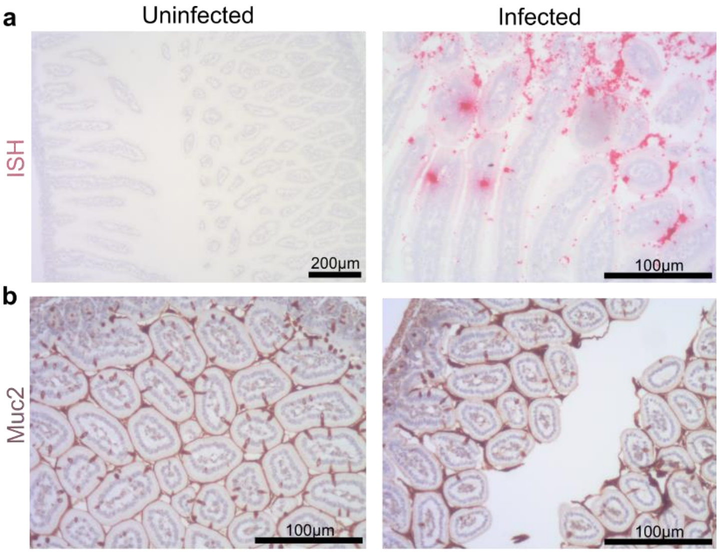

Supplementary Figure 8. Development of a neonatal model for murine astrovirus facilitated EPEC co-infections. a Hybridization of murine astrovirus-specific probes (red) to sections of small intestines collected at 7 dpi (corresponding to the day of harvest post-EPEC co-infection) from infected and uninfected 14-day-old C57BL/6 pups (n=4 animals/group). **b** Mucus thickness was visualized in serial sections from same tissues/animals by Muc2 staining (brown)**.** Data shown are from 2 replicate experiments.

Supplementary Table 1. Top differentially expressed genes among infected goblet cells relative to uninfected. Significance cut-off: FDR <0.05, Log_2 Fold change>0.5

Within infected animal		Between infected and uninfected animals	
**Increased**	**Decreased**	**Increased**	**Decreased**
Clps	*Rpl3*	*Isg15*	*Sec61b*
Ido1	*Rpl34*	*Irf7*	*Hspa5*
Odf2l	*Rps2*	*Ifi27l2b*	*Tpt1*
Clca1	*Sec61g*	*B2m*	*Rps24*
Mxd1	*Sec61b*	*Oasl2*	*Rpl3*
Zg16	*Tpt1*	*H2-K1*	*Rps20*
Muc2	*Rps7*	*Gcnt3*	*Tomm7*
S100a6	*Rps24*		*Rpl15*
Fer1l6	*Rpl27a*		*Rplp1*
Fcgbp	*Rpl14*		*Manf*
Tff3	*Rps3a1*		*Rps16*
H2-K1	*Rps12*		*Rpl35a*
	Rps20		*Hsp90b1*
	Rack1		*Rps8*
	Rpsa		*Rpl13*
	Rpl26		*Rps23*
	Rpl13		*Rplp0*
	Serf2		*Rpl34*
	Ostc		*Pdia3*
	Rps8		*Phb*

Supplementary Table 2. Mucus-associated bacteria identified by 16S sequencing

Taxa
Bacteria;Firmicutes;Clostridia;Clostridiales;Ruminococcaceae;Ruminococcus
Bacteria;Firmicutes;Bacilli;Lactobacillales;Streptococcaceae;Streptococcus;Streptococcus_oralis
Bacteria;Bacteroidetes;Bacteroidia;Bacteroidales;Bacteroidaceae;Bacteroides
Bacteria;Bacteroidetes;Bacteroidia;Bacteroidales;Bacteroidaceae;Bacteroides;Bacteroides_thetaiotaomicron
Bacteria;Firmicutes;Bacilli;Lactobacillales;Lactobacillaceae;Lactobacillus
Bacteria;Actinobacteria;Actinobacteria;Bifidobacteriales;Bifidobacteriaceae;Bifidobacterium
Bacteria;Verrucomicrobia;Verrucomicrobiae;Verrucomicrobiales;Akkermansiaceae;Akkermansia
