# Supplementary material for: Astrovirus infects actively secreting goblet cells and alters the gut mucus barrier
Source: Nat Commun. 2020 Apr 29;11:2097. doi: 10.1038/s41467-020-15999-y (PMC7190700; doi:10.1038/s41467-020-15999-y)
Supplement: Supplementary file 3 — Reporting Summary [file 41467_2020_15999_MOESM3_ESM.pdf]

nature research

Corresponding author(s): Stacey Schultz-Cherry

Last updated by author(s): Mar 21, 2020

Reporting Summary

Nature Research wishes to improve the reproducibility of the work that we publish. This form provides structure for consistency and transparency in reporting. For further information on Nature Research policies, see Authors & Referees and the Editorial Policy Checklist .

Statistics

For all statistical analyses, confirm that the following items are present in the figure legend, table legend, main text, or Methods section.

n/a	Confirmed	
☐	☒	The exact sample size (n) for each experimental group/condition, given as a discrete number and unit of measurement
☐	☒	A statement on whether measurements were taken from distinct samples or whether the same sample was measured repeatedly
☐	☒	The statistical test(s) used AND whether they are one- or two-sided<br>*Only common tests should be described solely by name; describe more complex techniques in the Methods section.*
☐	☒	A description of all covariates tested
☐	☒	A description of any assumptions or corrections, such as tests of normality and adjustment for multiple comparisons
☐	☒	A full description of the statistical parameters including central tendency (e.g. means) or other basic estimates (e.g. regression coefficient) AND variation (e.g. standard deviation) or associated estimates of uncertainty (e.g. confidence intervals)
☐	☒	For null hypothesis testing, the test statistic (e.g. F, t, r) with confidence intervals, effect sizes, degrees of freedom and P value noted<br>*Give P values as exact values whenever suitable.*
☒	☐	For Bayesian analysis, information on the choice of priors and Markov chain Monte Carlo settings
☒	☐	For hierarchical and complex designs, identification of the appropriate level for tests and full reporting of outcomes
☒	☐	Estimates of effect sizes (e.g. Cohen's d, Pearson's r), indicating how they were calculated

Our web collection on statistics for biologists contains articles on many of the points above.

Software and code

Policy information about availability of computer code

Data collection	No software was used for data collection.
Data analysis	10x gene expression data were first processed using Cell Ranger (v3.0.2, 10x Genomics). Seurat (v3.0.0.900) was then used for downstream analysis and data visualization. Illumina MiSeq paired-end reads for 16S metagenomic sequencing analysis were run through DADA2 pipeline (v1.10.1) to correct sequencing errors and determine amplicon sequence variants (ASVs) which represent original 16S rRNA gene amplicons in the samples. The ASVs were then used to recruit full-length 16S rRNA gene sequences from Ribosomal Database Project release 16.0 to construct a phylogenetic reference dataset from which a reference tree was built. The amplicon sequences were then placed onto the reference tree using the pplacer tool (v1.1.alpha19). Image J (v1.51u) was used to measure mucus thickness from images.

For manuscripts utilizing custom algorithms or software that are central to the research but not yet described in published literature, software must be made available to editors/reviewers. We strongly encourage code deposition in a community repository (e.g. GitHub). See the Nature Research guidelines for submitting code & software for further information.

Data

Policy information about availability of data

All manuscripts must include a data availability statement. This statement should provide the following information, where applicable:

- Accession codes, unique identifiers, or web links for publicly available datasets
- A list of figures that have associated raw data
- A description of any restrictions on data availability

Single-cell RNA sequencing and 16S metagenomic sequencing data that support the findings of this study have been deposited in NCBI BioProject with the primary accession code PRJNA573959. Reference murine astrovirus genome is available in GenBank accession JX544744 .1. All other data is available in the main text, supplementary materials, or Source Data file.

October 2018

Field-specific reporting

Please select the one below that is the best fit for your research. If you are not sure, read the appropriate sections before making your selection.

☒ Life sciences ☐ Behavioural & social sciences ☐ Ecological, evolutionary & environmental sciences

For a reference copy of the document with all sections, see nature.com/documents/nr-reporting-summary-flat.pdf

Life sciences study design

All studies must disclose on these points even when the disclosure is negative.

Sample size	No sample size calculations were performed, but all attempts at replication were successful or had repeated internal/longitudinal measures to ensure robustness. Animal group sizes were comparable across experimental groups and the total n used for each experiment was balanced with the use of living organisms in the absence of an alternative method of investigation. For these reasons, a sample size of n=5 was chosen for initial experimental studies. Housing of animals in our facility allows for a maximum of 5 animals/cage, which meant that groups of at least 2 and up to 5 animals would yield consistent data across replicate experiments and the amount of socializing and sharing of fecal microbiota, including virus shed, would be comparable.
Data exclusions	On Day 7, mouse 3 feces data was discarded due to a failed sequencing run that produced too few reads for 16S sequencing analysis (Figure S5a).
Replication	The number of replicates is stated for each experimental procedure and were combined for analysis. Representative images reflect the average for each study group. All replicate experiments were successful.
Randomization	All mice were age-matched. We previously showed a lack of sex differences in murine astrovirus infection and thus, a mixture of male and female mice were used.
Blinding	Blinding was not relevant to this study as it did not include clinical information or subjective measurements, such as behavior. For histology, the same regions within the intestine were examined for appropriate comparisons between groups.

Reporting for specific materials, systems and methods

We require information from authors about some types of materials, experimental systems and methods used in many studies. Here, indicate whether each material, system or method listed is relevant to your study. If you are not sure if a list item applies to your research, read the appropriate section before selecting a response.

Materials & experimental systems

n/a	Involved in the study	
☐	☒	Antibodies
☒	☐	Eukaryotic cell lines
☒	☐	Palaeontology
☐	☒	Animals and other organisms
☒	☐	Human research participants
☒	☐	Clinical data

Methods

n/a	Involved in the study	
☒	☐	ChIP-seq
☒	☐	Flow cytometry
☒	☐	MRI-based neuroimaging

Antibodies

Antibodies used	Ghost Dye Violet 510 (Tonbo, #13-0870-T500), PE anti-mouse EPCAM (clone G8.8, Biolegend, #118206), BV785 anti-mouse CD45.2 (clone 104, Biolegend, #109839) were used to sort out epithelial cells from single-cell suspensions generated after homogenizing duodenal tissues. Anti-mouse DCLK1 (abcam, #ab31704) and anti-mouse Muc2 (GeneTex, #GTX100664) were used for immunohistochemistry. Murine IL-4 (Sigma, #I1020), functional grade purified anti-mouse IL-4 (clone 11B11, eBioscience, #`6-7041-85), and murine IL-13 (Sigma, #I1896) were used in vivo.
Validation	Biolegend performs quality control testing by immunofluorescent staining with flow cytometric analysis. In addition, cells sorted from after staining with anti-mouse EPCAM and CD45.2 were confirmed to be epithelial-derived based on single-cell RNA sequencing. The anti-mouse DCLK1 antibody was validated by abcam using a knockout animal, while the anti-mouse Muc2 from GeneTex was validated by alcian blue or periodic-acid Schiff staining that similarly stain for secretory epithelial cells. Validation of IL-4 and IL-13 was not performed for thus study, but its effects on the proximal small intestinal goblet cells was visualized using alcian blue. Sigma validates both antibodies using proliferation assays using mouse HT-2 cells (IL-4) and human TF-1 cells (IL-13).

Animals and other organisms

Policy information about studies involving animals; ARRIVE guidelines recommended for reporting animal research

Laboratory animals	Male and female 8-week-old and 7-day-old C57BL/6 mice were used for experiments. Animals were kept under barrier conditions to keep them specific pathogen free. All cages, food, bedding, water bottles, and supplies were sterilized in bulk autoclaves. Animals were maintained in microisolation caging and cage changes were performed under a change station or Class 2A biological safety cabinet. The animal rooms are kept under negative pressure relative to the corridor with a ventilation system designed to supply 100% fresh outside air which has been heated or cooled and HEPA filtered. The system is designed to provide 10 or more draft-free air-exchanges per hour to animal rooms. The temperature inside the animal room during these experiments averaged 68 degrees with 45% humidity.
Wild animals	The study did not involve wild animals.
Field-collected samples	The study did not involve samples collected from the field.
Ethics oversight	All animal experiments were approved by the St. Jude Children's Research Hospital (St. Jude) Institutional Animal Care and Use Committee (protocol 570). St. Jude is fully accredited by the Association for the Assessment and Accreditation of Laboratory Animal Care International (AAALAC-I) and has an approved Animal Welfare Assurance Statement on file with the Office of Laboratory Animal Welfare (A3077-01). These guidelines were established by the Institute of Laboratory Animal Resources and were approved by the Governing Board of the U.S. National Research Council.

Note that full information on the approval of the study protocol must also be provided in the manuscript.
